# Prognostic Value of *CD200* Expression and Soluble CTLA-4 Concentrations in Intermediate and High-Risk Myelodysplastic Syndrome Patients

**DOI:** 10.31557/APJCP.2020.21.8.2225

**Published:** 2020-08

**Authors:** Salah Aref, Mohamed El Agdar, Ahmed El Sebaie, Tarek Abouzeid, Mohamed Sabry, Lamiaa Ibrahim

**Affiliations:** 1 *Hematology Unit, Clinical Pathology Department, Faculty of Medicine, Mansoura University, Egypt. *; 2 *Hematology Unit, Mansoura University Oncology Center, Faculty of Medicine, Mansoura University, Egypt. *; 3 *Clinical Hematology unit, Mansoura University Oncology Center, Faculty of Medicine, Mansoura University, Egypt. *

**Keywords:** MDS, CD200, CTLA-4, outcome

## Abstract

**Objective:**

This study was designed in order to identify the prognostic relevance of *CD200* expression and soluble Cytotoxic T-lymphocyte antigen-4 (CTLA-4) levels in myelodysplastic syndrome (MDS) patients.

**Methods:**

The study included 57 MDS (37 intermediate and 20 high risk) patients and 10 controls. For all of included patients; *CD200* expression was identified by flowcytometry on CD33 positive cells and soluble CTLA-4 (CD152) concentration was determined by ELISA.

**Results:**

*CD200* positive expression was detected in 32/57 (56.1%) of MDS cases, the mean serum CTLA-4 concentrations were significantly higher in MDS patients as compared to controls (P<0.01). Significant association between high *CD200* positive expression; high CTLA-4 concentration levels and MDS risk stages being higher in high risk MDS group as compared to intermediate risk one (P < 0.01). After 36-month follow-up; the subgroup of MDS patients with high expression of *CD200*; and high serum CTLA-4 concentrations showed high death rate and high frequency of acute myeloid leukemia transformation.

**Conclusions:**

*CD200* positive expression could be considered as a new prognostic marker for risk stratification of MDS patients. *CD200* expression may exert its effect through upregulation of CTLA-4.

## Introduction

Myelodysplastic syndrome (MDS) is a complex clonal hemopoietic progenitor cell disorders that result from the evolution of aberrant clones and is characterized by an ineffective hematopoiesis and frequent cell apoptosis in the bone marrow (BM) and manifested by blood cytopenia’s (Montalban-Bravo and Garcia-Manero., 2018). Pathophysiological causes of this disease are diverse including genetic abnormalities within myeloid progenitors, altered epigenetics, changes in the bone marrow microenvironment and the immune disorder. Since the mechanisms contributing to immune pathophysiology remain elusive in MDS patients, a search for predictive biomarkers is critically needed (Aleshin A and Greenberg., 2018).

The clinical course of Myelodysplastic Syndromes (MDS) are very heterogeneous, with a wide diversity in patient’s outcome. Whilst near one third of MDS progress to AML; the other two thirds transformed from low risk into high risk MDS (Garcia-Manero 2015); The pathophysiology of MDS is not established and poorly defined. Several hypotheses were postulated which includes immunological derangement. Due to the heterogeneity in the morphology and variability in the patient’s outcome; it is difficult to classify and to predict the MDS patient’s outcome (Chen et al., 2017).

*CD200* is one of the immunoglobulin superfamily proteins. *CD200* receptor expression is restricted to myeloid cells. It composed of 2 extracellular domains and one small intracellular domain. *CD200* tumor expression down regulate Th1 cytokine production when cocultured with allogenic leukocytes (Chokr et al,.2018; Kawasaki et al,. 2018).

Cytotoxic T-lymphocyte associated antigen-4 (CTLA-4) is a costimulatory receptor transducing a potent inhibitory signal to the T cell and thus limiting immune responses (Kawasaki et al, 2018). Accumulating evidence revealed that CTLA-4 gene have a significant role for autoimmune disease. The soluble forms is produced by alternatively splicing of mRNA produce a soluble form lack derived from lacking of transmembrane sequence (Simone et al., 2012) named sCTLA-4. Low levels of sCTLA-4 were reported in the serum of normal human; on the other hand, elevated serum concentration levels were detected in many autoimmune disorders. A dual effect of sCTLA-4: the first is through inhibiting the secretion of IFN-γ, IL-2, IL-7, and IL-13 and activating the secretion of TGF-β and IL-10 (Oaks and Hallett 2000; Simone et al., 2014).

This study aimed to determine the prognostic relevance of *CD200* expression and soluble CTLA-4 in MDS patients.

## Materials and Methods

*Subjects and Methods*

This is a case control study; in which BM samples were obtained from 57 MDS patients (30 males; 27 females) with mean age 59.9±6.2 years and 10 controls (Refereed for hip bone replacement) (6 males; 4 females); mean age 60.3±6.6 years). Our study was conducted at Mansoura University hospitals, between January 2013 and December 2017 after taken informed consent. The diagnosis of MDS cases was based on cytomorphology findings of dysplasia in the bone marrow smears and bone marrow biopsy. Findings, cytogenetic studies, flow cytometry and genetics findings.

Exclusion criteria included patients with a history of autoimmune disease, HIV or solid organ transplantation or patients previously treated with chemotherapeutic agents or low risk MDS (very good and Good risk MDS).

The patients at diagnosis were classified according to WHO classification into Refractory cytopenia with multilineage dysplasia (n= 37); Refractory anemia with excess blast type I (n=8); Refractory anemia with excess blasts type II (n=12). According the Revised International Prognostic Scoring System (IPPS-R) for MDS (Zeidan et al., 2017); the patients were classified into 37 as intermediate risk and 20 as high risk MDS. Ten normal controls who refereed for hip bone replacement (6 males; 4 females; mean age 60.3±6.6 years) were included. Follow up for the included MDS patients for 3 year 13 cases were transformed to AML and 14 cases died during the first year follow up. Informed consents were obtained from all cases before inclusion in the study. The Revised International Prognostic Scoring System (IPPS-R) for MDS was used for stratification into prognostic subgroups (Zeidan et al., 2017).

*Therapy*

The selection of the therapy based on risk adapted policy which includes transfusion requirement; bone marrow blast cells percentage; cytogenetics; as well as mutational characteristics. The aim of the treatment is different according to IPPS-R in the low risk group the aim is to reduce the transfusion frequency and also to reduce the risk of AML transformation. While in the high-risk group the target is to extend the patient survival. The therapeutic lines include lenalidomide in the low risk group. Hemopoietic growth factor; Demethylating drugs like 5 azacytidine and decitabine in both low and high risk MDS; intensive chemotherapeutic agents as well as bone marrow transplantation in high risk group (Platzbecker., 2018)

*Flow Cytometric Immunophenotypic Studies*

Bone marrow samples were collected on EDTA tubes from MDS cases and controls. The analysis was done in the same day of sample collection. The gating strategy was done on CD45 (FITIC) positive cells; then sequential gating was done using CD33+*CD200*+ cells. Briefly; 5 μl of selected proper monoclonal antibodies to each tube containing 100 μl whole blood, incubated 20-30 min in dark at room temperature, washed with phosphate buffered saline (PBS) three times, and then added 300 μl PBS before analysis. Isotopic control using IgG1 and IgG2 on each sample was done to exclude auto fluorescence using BD FACS Canto II flow cytometer (BD Bioscience, San Jose, CA), and 50,000 events were collected in most cases wherever possible. The data analysis was done by software obtained from BD FACSDiva (BD Bioscience, San Jose, CA). 

*Cytogenetic analysis*

Conventional cytogenetic analysis was done through culture of bone marrow cells for 24 hours; the chromosomal finding was characterized according to International Human Chromosomes Nomenclature (ISCN, 1995).

*Serum sCTLA-4 levels by ELISA*

ELISA detection for serum sCTLA-4 concentration levels (Bender Medsystems, Milano, Italy), was carried out according to the included manufacturer instructions, briefly each sample was diluted 1: 10 and tested in triplicate. The standard deviation between the results of the 3 samples was less than 10% for each single result. The lowest threshold levels for kit sensitivity was 0.1 ng/ml. 

*Statistical analysis*

Data was analyzed using SPSS program version 16. Qualitative variables were presented as number and per cent. Chi-square or Fisher’s exact test was used for comparison between groups, as appropriate. Quantitative variables were tested for normality distribution using Shapiro test. Normally distributed variables were presented as mean ±SD and unpaired t-test was used for comparison between groups. Non-parametric variables were presented as median (minimum- maximum) and Mann-Whitney test was used for group comparison. Cox regression analyses were performed to calculate hazard ratios (HR) and associated 95% confidence interval (CI) for OS and MDS transformation to AML. In bivariate analysis we used age; Sex; *CD200* expression; CTLA-4 concentrations and MDS stage to predict AML transformation. While to predict survival among MDS patients we included the previously mention parameters in addition to AML transformation. The significant parameters in bivariate analysis were included in multivariate analysis. A P**<**0.05 was considered statistically significant

## Results

*The expression level of CD200 and Serum sCTLA-4 concentrations levels in MDS versus controls*

The *CD200* expression was identified on bone marrow cells expressing CD33 and compared to that in normal bone marrow. The frequency of *CD200* positive expression was detected in 32/57 (56.1%) of MDS cases; and no one of the control group was *CD200* positive. The CTL4 serum levels were significantly higher in the MDS group as compared to control group (P=0.001). Regarding the age and sex there was no significant differences in the *CD200* expression or serum CTLA-4 concentration levels (P>0.05) (Table 1).

*The CD200 expression and Serum sCTLA-4 concentration levels in MDS Stages*

Of the *CD200* positive cases; 12/40 (30.0%) were categorized as intermediate risk MDS and 15/17 (88.2%) were categorized as high risk MDS and the differences were statistically significant (P<0.001) (Table 2). The mean CTLA-4 serum concentration levels were significantly higher in high risk MDS group as compared to intermediate risk MDS group (P<0.01) (Table 2).

*Impact of CD200 expression and Serum sCTLA-4 concentration levels on MDS transformation to AML*

The MDS patients which transformed to AML showed high frequency of *CD200* positive expression and high concentration of serum CTLA-4 levels as compared to MDS cases that were not transformed to AML (P=0.001 for both) (Table 3).

*CD200 expression and serum CTLA-4 concentration in dead versus Living MDS patients*

After 36 months follow-up, we found that the *CD200* positive expression and CTLA-4 concentration levels were significantly higher in dead MDS patients’ group as compared to those in the living group (P<0.01; P=0.01 respectively) (Table 4). 

*Cox regression hazards ratio (HR) to predict AML transformation*

To calculate the HR for MDS on transformation to AML. We tested age; Sex; *CD200* expression; MDS stages; serum CTLA-4 concentrations in bivariate analysis. The analysis revealed that the parameters with high HR are *CD200* positive expression; CTLA higher concentration and advanced MDS stage. Moreover; in multivariate analyses including all parameters that proofed to be significant in bivariate analysis; the results reported that advanced MDS stage is the independent predictor of AML transformation [ARR (95%CI) 6.4 (1.1-38.6)] (P=0.04)] (Table 5).

*Cox regression hazards to predict patient’s outcome*

To calculate the HR for MDS on patient’s survival. We tested age; Sex; *CD200* expression; MDS stages; serum CTLA-4 concentrations and AML transformation in bivariate analysis. The analysis revealed that the parameters with high HR are *CD200* positive expression; CTLA higher concentration and advanced MDS stage and AML transformation. Moreover; in multivariate analyses including all parameters that proofed to be significant in bivariate analysis. The multivariable analysis reveal that the only significant variables is AML transformation [ARR (95%CI) 34.9 (7.4-64.7) (P<0.001) that predict patients’ outcome (Table 6).

**Table 1:** Comparison between Patients and Control in All Parameters

	MDS Cases (n=57)	Control (n=10)	Significance test
Sex			
Female	27 (39.2)	4 (40)	P>0.05
Male	30 (60.8)	6 (60)	
Age: Mean ±SD	54.3±6.8	54.3±6.1	P>0.05
*CD200*+ No (%)	27 (47.4)	0	P>0.05
CTLA-4 (ng/ml) Median (min-max)			
	6 (0.1-90)	0.1 (0-80)	P<0.001

FET, Fisher's exact test Z of Mann-Whitney test

**Table 2:** *CD200* Expression and CTLA-4 Concentration Levels in MDS Stages

	Intermediate risk (n=40)	High risk (poor and very poor) (n=17)	Significance test
*CD200+* No (%)	12 (30%)	15 (88.2)	P<0.001
CTLA-4 (ng/ml) Median (min-max)	5.0 (0.4-46)	35.0 (0.8-90)	P<0.001

KW, Kruskall-Wallis

**Table 3:** Comparison of Different Studied Parameters in MDS Patients Transformed to AML versus Those Not Transformed to AML

	MDS cases not transformed (n=44)	MDS cases transformed to AML (n=13)	Significance test
*CD200*+ No (%)	14.0 (31.8)	13.0 (100%)	P<0.001
CTLA-4 Median (min-max)	3.5 (0.1-77)	34 (1.3-90)	P<0.001

**Table 4:** Comparison of the Different Parameters in Died MDS Patients as Compared to Live Ones

	Alive (n=43)	Died (n=14)	Significance test
*CD200*+ No (%)	16.0 (36.4%)	11.0 (84.6%)	P<0.001
CTLA-4 Median (min-max)	5.0 (0.1-77)	33.0 (0.4-90)	P<0.01

FET, Fisher's exact test; MET, Monte Carlo Exact test; Z of Mann-Whitney test

**Table 5:** Cox Regression Analysis to Predict AML Transformation in MDS Patients

	MDS (n=44)	Transformed AML (n=13)	*P*-value	CRR (95%CI)	Logistic regression	
					P-value	ARR (95%CI)
Age: Mean ±SD	53.9±6.6	56.7±7.6	>0.05	2.2 (0.6-7.2)	-	-
Sex					-	
Female (n=27)	15 (55.6)	12 (44.4)	>0.05	1.4 (0.1- 18.2)		
Male (n=30)	17 (56.7)	13 (43.3)				
*CD200*		0		7.8		
-ve (n=30)	30 (100)	13 (48.1)	<0.001	(1.9-32.8)		
+ve (n=27)	14 (51.9)					
CTLA-4						
Median (min-max)	3.5 (0.1-77)	34 (1.3-90)	<0.001	8.4 (3.1-32.6)		
MDS stage:						
Intermediate (n=40)	40.0 (100%)	0 (0%)		9.2 (2.8-30.3)	0.04	6.4 (1.1-38.6)
Vs			<0.001			
High risk (n=17)	4.0 (23.5%)	13 (76.5%)				

CRR, Crude relative risk; ARR, adjusted relative risk; CI, Confidence interval; Variables included in logistic regression are: CTLA-4; AML and MDS stage; All parameters were included in bivariate analysis. The variables which are significant in bivariate analysis were included in the multivariate analysis. In multivariate analysis, ARR and their 95% CI only for the significant independent predictor is presented in the Table.

**Table 6:** Cox Regression Analysis to Predict Patient’s Outcome

	Alive (n= 43) N (%)	Died (n=14) N (%)	*P*- value	CRR (95%CI)	Logistic regression	
					*P*-value	ARR (95%CI)
Age: Mean ±SD	53.9±6.8	56.3±6.9	>0.05	1.8 (0.4-6.6)		
Sex						
Female(n=27)	23 (90.9)	4 (9.7)	>0.05	2.2 (0.6-7.2)		
Male (n=30)	20 (79.2)	10 (20.8)				
*CD200*+ (n=17)	6 (35.3%)	11 (64.7%)	<0.001	5.8 (1.4-25.0)		
*CD200*- (n=30)	27 (90%)	3 (10.0%)				
CTLA-4						
Median (min-max)	5 (0.1-77)	33 (0.4-90)	<0.01	7.3 (1.7-30.7)		
MDS stage						
Intermediate (n=40)	35 (87.5%)	5 (12.5%)	<0.001	6.2 (2.1-18.1)		
Vs						
High risk (n=17)	8 (47.0%)	9 (52.9%)				
MDS transformed to AML						
No (n=44)	41 (93.1%)	3 (6.8%)	<0.001	11.4 (4.1-31.6)	<0.001	34.9 (7.4-64.7)
Yes (n=13)	2 (15.4%)	11 (78.6)				

CRR, Crude relative risk; ARR, adjusted relative risk; CI, Confidence interval; Variables included in logistic regression are: CD4, CD34, CTLA-4, AML and MDS stage; All parameters were included in bivariate analysis. The variables which are significant in bivariate analysis were included in the multivariate analysis. In multivariate analysis, ARR and their 95% CI only for the significant independent predictor is presented in the table.

## Discussion

The immune dysregulation is one of the postulated theories to explain the pathogenesis of MDS. The previous studies reported that among the immune inhibitory mediators are *CD200* and CTLA-4. Herein, we assessed these 2 inhibitory factors and their impact on MDS evasion from the immune system (Rudd 2008; Bisgin et al,. 2019). Despite that the over-expression of *CD200* has been implicated in the pathogenesis of solid tumors, hematological malignancies and its expression is indicative of poor prognosis; very few studies have investigated its accuracy in MDS patients; which substantially differ from them. 

In this study *CD200* positive expression was significantly higher in MDS patients as compared to controls. This finding was in parallel with that reported by Chen et al (2017). In previous studies, *CD200* was reported to take part in several cancers. For instance, Tiribelli et al (2017) discovered the expression of *CD200* in acute myeloid leukemia and proved that its antigen expression was related to the poor prognosis of this disease. The over-expression of *CD200* is linked with expansion of suppressive Treg cells and plays a vital role in the progression of multiple myeloma (Aref et al., 2017). So far, Several reports concluded that *CD200* expression is also associated with the progression of various solid cancers, such as bladder cancer, lung cancer, breast cancer prostate carcinoma, cutaneous squamous cell carcinoma and acute myeloid leukemia (Kretz-Rommel et al., 2007; Lawlor et al., 2019; Aref et al 2020).

After 24 months follow up of the MDS patients with high *CD200* expression and high sCTLA-4 concentration showed high number of deaths; a high Leukemic transformation. These finding was in agreement with the findings reported by Chen et al., (2017).

Our results are in agreement with previous ones that reported that not only MDS patients display over-expression of *CD200* on CD33+ bone marrow cells, but also *CD200* expression was significantly associated with high risk MDS subtype being higher in high risk group as compared to intermediate risk group. Moreover, the findings in the current study demonstrated that *CD200* positive expression was an independent predictor of MDS transformation to acute myeloid leukemia. These findings were in parallel with that reported in the previous studies which confirmed the prognostic relevance of high *CD200* expression in MDS patients (Tiribelli et al., 2017; Gorczynski et al .,2017; Coles et al ., 2012). 

CTLA- 4 serum concentration levels were significantly higher in MDS patients as compared to controls and in high risk MDS group as compared to intermediate risk group. Furthermore; MDS patients with high sCTLA-4 was associated with high frequencies of deaths; AML transformation. No previous studies reported similar findings. This could be explained on the basis that the CTLA-4 molecule downregulate T-cell activation to maintain peripheral tolerance, and can be exploited by tumors to induce an immunosuppressive state that allows the tumors to grow and develop instead of being eliminated by the immune system (Buchbinder and Desai 2016; Elizabeth et al., 2016). In addition, Simone et al., (2012) stated that sCTLA-4 modulate the immune response through a dual effect; the first one through down regulation the secretion of IFN-γ, IL-2, IL-7, and IL-13 and the second one upregulates the secretion of TGF-β and IL-10 (Kawasaki et al.,2008; Oaks et al., 2000).

To test whether *CD200* was involved in the progression of MDS. We estimate both survival and risk of AML transformation at multiple time points during the natural course of MDS. After 24-month follow-up, of patients with MDS that transformed to AML was significantly higher in cohort of MDS patients with Positive *CD200* expression. This finding was in agreement with that reported by Chen et al., (2017).

In conclusion, *CD200* over expression and CTLA-4 higher concentration were detected in MDS. *CD200* positive expression and higher sCTLA-4 concentration in MDS patients at diagnosis could be considered as a new prognostic marker for risk stratification. *CD200* may exert its effect through upregulation of CTLA-4. These data suggest that simultaneous blockade of both CTLA-4 and *CD200* pathways might have a role treatment of MDS through up regulation of antitumor immune responses.

*Ethical aspects*

This study was approved by the local Institutional Ethics Committee. Written informed consent from the participants will be obtained by the principal investigator prior to recruitment. The authors declare that there is no conflict of interest.
